# The evolution of PFAS epidemiology: new scientific developments call into question alleged “probable links” between PFOA and kidney cancer and thyroid disease

**DOI:** 10.3389/fpubh.2025.1532277

**Published:** 2025-04-09

**Authors:** Catie Boston, Stella Keck, Avery Naperala, Justin Collins

**Affiliations:** Roux, Burlington, MA, United States

**Keywords:** per- and polyfluoroalkyl substances, perfluorooctanoic acid, PFOA, kidney cancer, thyroid disease, general causation

## Abstract

The growing body of litigation alleging bodily injury from per- and polyfluoroalkyl substances (PFAS) exposure has put a spotlight on the available scientific literature regarding potential human health impacts, and the various data gaps within the literature. This review assesses the evolution of epidemiological findings for perfluorooctanoic acid (PFOA), a PFAS compound. In 2012, the C8 Science Panel published a series of reports determining “probable links” for certain health outcomes (including kidney cancer and thyroid disease); it was the first major research effort investigating potential adverse health effects following exposure to PFOA. At that time, there were only a handful of available studies investigating human effects (i.e., epidemiological studies). Now, over a decade later, the epidemiological body of literature for PFOA has grown substantially. As is the nature of evolving science, the additional research has spotlighted important improvements in exposure classification, confounding control, and statistical methods that strengthen more recent scientific investigations. As the body of epidemiological literature for PFAS health effects grows and evolves with improved methodology, the original C8 Science Panel’s conclusions have not been supported by more recent investigations. Within the context of general causation, while gaps remain in the body of research, more recent epidemiological findings support that there is no causal relationship between PFOA exposure and kidney cancer or thyroid disease.

## Introduction

Per- and polyfluoroalkyl substances (PFAS) are a group of thousands of man-made chemicals that are utilized in a variety of commercial and industrial applications (1). Perfluorooctanoic acid (PFOA), also referred to as “C8,” is historically one of the most extensively produced and used PFAS, with production of PFOA dating back to the 1940s (2, 3). The nickname C8 derives from the chemical structure of PFOA, which is comprised of a seven-carbon-long perfluoroalkyl chain with a carboxylic acid group on the end that totals eight connected carbons (4); of note, there are other PFAS with eight carbons (e.g., PFOS). Due to their widespread use and extraordinary stability, PFAS as a group are widely distributed and highly persistent in the environment.

While regulatory developments regarding PFAS have dominated the news in recent years, a growing body of litigation alleging bodily injury from PFAS exposure is gaining momentum. The first wave of bodily-injury litigation alleging direct exposure to PFAS from firefighting foam has produced nearly 10,000 lawsuits filed in federal multi-district litigation (MDL) court.^1^ It’s expected that the number of bodily-injury lawsuits alleging direct or indirect exposure to PFAS will only grow, with the outcome of the MDL either adding fuel to the fire or making future claims more tenuous.

With a focus on the various allegations of exposure and bodily injury asserted by plaintiffs against a myriad of defendants, the scientific community has become more active in recent years investigating issues of scientific causation and examining if there is a definitive link between exposure to PFAS compounds and disease. This evolution of PFAS science is likely to have a profound impact on upcoming bellwether test trials in the MDL litigation and may shape how bodily-injury cases are adjudicated in state and federal courts around the country. Although there are thousands of PFAS, this review focuses on PFOA as a case-study.

Initial PFAS health effects research stems from a landmark case in West Virginia that investigated possible links between PFOA and certain cancers and other diseases. In February 2005, the West Virginia Circuit Court approved a class action settlement related to releases of PFOA from DuPont’s Washington Works. A centerpiece of the settlement was the creation of a Science Panel (the “C8 Science Panel”), appointed by both parties, with the goal of determining whether there was a “probable link” between exposure to PFOA and disease within the community. In 2012, the C8 Science Panel determined that there was a “probable link” between exposure to PFOA in drinking water and high cholesterol, ulcerative colitis, thyroid disease, testicular cancer, kidney cancer, and pregnancy-induced hypertension in the class. This review focuses on research issued after 2012 specific to two of those diseases: kidney cancer and thyroid disease. The C8 Science Panel evaluated the epidemiological evidence available at the time (and generated conclusions as part of the settlement), without the benefit of current understanding of nuances of PFAS science, or the benefit of a more mature epidemiological database.

While the focus of the C8 Science Panel was only on PFOA, the importance of utilizing the evolving science described above holds true for other PFAS (such as PFOS, PFHxS, PFHxA, PFNA, PFBS, etc.). Moreover, this examination of the science highlights the distinction and nuance between legal rulings and settlements and true scientific causation—dispelling any assertions that legal rulings equate to “settled science.” In this review, we examine the epidemiological literature to assess the potential associations between exposure to PFOA and kidney cancer or thyroid disease in order to distinguish between legal settlements and scientific causation.

### Findings of the C8 Science Panel

The 2012 C8 Science Panel, chosen jointly by parties involved in a PFOA-specific legal settlement, was one of the first significant efforts that investigated potential health effects associated with exposure to PFOA. Conducted from 2005 to 2013, the C8 Science Panel carried out a series of exposure and health studies in the Mid-Ohio Valley communities impacted by high concentrations of PFOA, or “C8” to drinking water sources from the DuPont’s Washington Works plant in West Virginia (36). The Washington Works facility historically manufactured Teflon, and discharges from the plant from approximately 1984 to 2004 impacted the Ohio River, the principal source of public drinking water for many residents of Ohio and West Virginia living in the vicinity of the Washington Works plant.

The studies in the C8 Science Panel were the largest epidemiological studies of PFOA conducted at the time of publication. From 2005 to 2006, the C8 Health Project enrolled 69,030 participants for 11 distinct epidemiological studies. In 2012, the C8 Science Panel published “probable link” reports digesting the results of the studies considered; a “probable link” is a determination specific to the community involved in the settlement, and was defined as: “given the available scientific evidence, it is more likely than not that among class members a connection exists between PFOA exposure and a particular human disease” (36). The Panel’s conclusions were specific to high exposures of PFOA, included studies that looked at association rather than causation, and were determined under the context of litigation—in other words, “probable links” were conservatively determined not using traditional general causation methodology. Assessment of general causation includes evaluation of biological plausibility via mechanistic toxicological understanding, as well as consideration of toxicological investigations of dose–response in highly exposed laboratory animals. The C8 Science Panel concluded that there was a “probable link” to PFOA exposure for the following categories: diagnosed high cholesterol, ulcerative colitis, thyroid disease, testicular cancer, kidney cancer, and pregnancy-induced hypertension. The Panel did not find “probable links” with exposure to PFOA in drinking water and the other conditions investigated (e.g., liver disease, diagnosed hypertension, coronary artery disease, chronic kidney disease, osteoarthritis, and many others) (36).

Since the initial publication of the results from the C8 Science Panel, there continues to be extensive research investigating PFOA and PFAS generally, with an exponential increase in publications over the last decade (5). Major regulatory and authoritative bodies have released their own reviews regarding PFAS and their potential health effects, including the United States Environmental Protection Agency (USEPA), International Agency for Research on Cancer (IARC), and Agency for Toxic Substances and Disease Registry (ATSDR), among others. These reviews assessed scientific literature beyond epidemiological evidence.

Since 2012, the authors from the C8 Science Panel themselves contributed to this growing body of literature, collectively authoring over 40 follow-up studies (36). Beyond PFOA, extensive research has been conducted investigating PFAS as a group, as well as differences across individual PFAS compounds. The available evidence indicates that there are differences in toxicity depending on the specific PFAS in question (such as relative potencies, differences in mechanism of toxicity, differences in elimination half-life, etc.) (37). In the ideal assessment of the potential health effects from exposure to PFAS, there would be a distinction between exposure to individual compounds rather than exposure to mixtures of PFAS. This is difficult in practice, however, as the general population may be exposed to mixtures of different PFAS that are found in a variety of different media in the environment (37).

In general, scientific understanding of chemicals evolves over time as investigators explore hypotheses in different populations, exposure settings, and experimental systems. Knowledge gleaned from one investigation is leveraged by other scientists to refine protocols and hypotheses related to exposure assessment and to understand potential manifestation of disease in humans. For example, improved environmental sampling techniques and more sophisticated analytical tools allow for more accurate exposure quantification, or epidemiologists may discover an important confounder that, once controlled for, attenuates observed associations (i.e., when other factors that can potentially explain the relationship are included in the statistical model, the result becomes no longer statistically significant—a potential causal relationship does not exist).

Reproducibility of findings is one of the most important parts of the scientific process; consistent findings across multiple high-quality investigations is a core tenet of the weight of evidence approach that allows for the understanding of a potential causal relationship (e.g., Bradford Hill Criteria) (6). To establish that exposure to an agent causes a health outcome, a variety of evidence is used in tandem to demonstrate biological plausibility through an evaluation of toxicokinetic studies, studies investigating mechanistic or structural capability, and dose–response assessments in animal bioassays. It is beyond the scope of this analysis to describe in detail the methodology to assess general causation, and this analysis does not attempt to perform a general causation assessment. Rather, this review examines the state of the art in epidemiological science regarding PFOA and certain health outcomes and applies that evidence to distinguish between legal rulings and settlements and epidemiological findings supporting scientific causation. While a full general causation analysis would incorporate mechanistic and animal evidence, this analysis is limited to human epidemiological studies that evaluate doses relevant to real-world exposures, which are the most useful studies when evaluating causation.

## Methodology

This narrative review evaluates epidemiological investigations of PFOA over time to highlight the dynamic evolution of scientific understanding. This article is not a systematic review. The review is limited to PFOA exposure and kidney cancer and thyroid disease in humans. Relevant studies were identified through PubMed and Google Scholar using the search terms “PFOA” and “kidney cancer,” “PFOA” and “thyroid disease,” “perfluorooctanoic acid” and “kidney cancer” and “perfluorooctanoic acid” and “thyroid disease”; additional search terms included specific types of thyroid disease such as “hypothyroidism” and “hyperthyroidism.” The review focused on human studies that specifically evaluated PFOA exposure and included effect estimates regarding kidney cancer or thyroid disease (n = 14 and n = 11, respectively). Study quality is reviewed and discussed in the review qualitatively. No formalized scoring criteria were utilized; however, sample size, methodology, and adjustment for confounders are all considered as described herein. Existing agency reviews were utilized to cross-reference for critical studies.

## Evolution of epidemiological science since the C8 Panel

To demonstrate the evolution of the PFOA epidemiological literature, we discuss two health outcomes identified by the C8 Science Panel as having a “probable link” with PFOA exposure: (1) kidney cancer, and (2) thyroid disease. This review focused on two outcomes for the sake of brevity, and because both health outcomes have been studied extensively since the 2012 C8 Panel, whereas other outcomes have not been studied to the same degree. As discussed below, for some health outcomes, the more recently conducted epidemiological studies clearly support no association despite the findings of the 2012 C8 Panel, while for other health outcomes, the evidence is equivocal. Notably, there are important study design considerations unique to PFAS as a group that complicate interpretation of certain studies such as adequate control of confounders, the importance of exposure classification, and choice of statistical methods.

### Kidney cancer

In 2012, the C8 Science Panel determined that the available epidemiologic data presented sufficient evidence to conclude that there was a “probable link” between PFOA exposure and kidney cancer (36). The C8 Panel considered the following studies related to PFOA exposure and kidney cancer:

- Leonard et al. (7) is an independent mortality study conducted at the DuPont Washington Works plant. The study reported an elevated rate of kidney cancer mortality, but the standardized mortality rate (SMR) did not reach statistical significance (SMR = 1.81, 95% CI = 0.94–3.16).
- Steenland and Woskie (8) is a C8 Science Panel study that extended the follow-up time of Leonard et al. (7). The study authors reported increased kidney cancer mortality in the top exposure quartile in a lagged analysis (SMR = 3.67, 95% CI = 1.48–7.57); however, the sample size of kidney cancer deaths is small (n = 11) and may not be generalizable beyond the study population. With all quartiles combined, there was no significant association between PFOA exposure and kidney cancer mortality (SMR = 1.28, 95%CI = 0.66–2.24).
- Barry et al. (9) is an investigation conducted by the C8 Science Panel in 2012 that was later published in 2013. This study focused on residential exposure to PFOA in drinking water for residents that lived in the community surrounding the Washington Works plant stratified into occupational and community groups. The study authors reported no increased risk of kidney cancer when looking at a lagged analysis of the whole cohort [Hazard Ratio (HR) = 1.09, 95% CI = 0.97–1.21] or in any quartile of cumulative PFOA exposure [Quartile 4 (Q4) HR = 1.43, 95% CI = 0.76–2.69]. In the 10-year lagged stratified analysis, there was no increase in kidney cancer in the occupational (HR = 0.99, 95% CI = 0.67–1.46) or community (HR = 1.11, 95% CI = 0.96–1.29) groups. An increased risk of kidney cancer was observed in the highest quartile of PFOA exposure in the unlagged analysis of the community group (HR = 2.04, 95% CI = 1.07–3.88); however, the association was attenuated in their 10-year lagged analysis (HR = 1.5, 95% CI = 0.72–3.13).
- Vieira et al. (10) was also considered in the “probable link” report (but was not published until 2013). This study examined serum concentrations of PFOA among residents of multiple communities exposed to PFOA in drinking water in the vicinity of the Washington Works plant. Looking at the entire study population, odds of kidney cancer diagnosis were significantly increased in the “high” modeled serum PFOA quartile [Adjusted Odds Ratio (AOR) = 2.0, 95% CI = 1.3–3.2] and borderline significantly increased in the “very high” modeled serum PFOA quartile (AOR = 2.0, 95% CI = 1.0–3.9). In their analysis of individual communities, however, the authors reported a statistically significant increase in odds of kidney cancer in only one out of six communities (Tuppers Plains; AOR = 2.0, 95% CI = 1.3–3.1).

Despite the inconclusive results, the 2012 C8 panel nonetheless concluded,

“For *kidney cancer*, the worker mortality study conducted by the Science Panel showed a higher risk in the most highly exposed group compared to lower exposure groups among the workforce, but the risks were not elevated compared to the US population. In the cohort study, there was a gradient of increasing risk with increasing exposure but most strongly in the analyses that included exposure up to the time of diagnosis. When the 10 years of exposure prior to diagnosis was excluded, the association was less evident. No association was seen in the prospective analysis of cohort data, although the latter is limited by small numbers. In the geographic study some results suggested an increasing risk of kidney cancer with increasing exposure and others did not. The science panel considers that the excesses observed indicate a probable link between PFOA and kidney cancer.”

Since the C8 report, there have been four additional primary studies (11–14), one pooled study (15), which combined data from Barry et al. (9) and Shearer et al. (12), and a meta-analysis (16), not to mention multiple review papers and studies investigating general PFAS exposure (not specific to PFOA). The primary studies are detailed below.

- Raleigh et al. (11) is a mortality cohort study that investigated occupational exposure to ammonium perfluorooctanoate (APFO) among workers at 3 M’s PFOA production plant in Cottage Grove, MN. The authors reported no statistically significant association between exposure and kidney cancer when examining the total population (SMR = 0.53, 95% CI = 0.20–1.16), or when stratifying exposure to high and low groups (Q1–Q2 HR = 0.38, 95% CI = 0.11–1.23, Q3–Q4 HR = 0.39, 95% CI = 0.11–1.32).
- Shearer et al. (12) is a nested case–control study conducted in the Prostate, Lung, Colorectal and Ovarian Cancer Screening Trial (PLCO). The study controlled for multiple confounders and reported that when the exposure was evaluated continuously, the association between PFOA and kidney cancer was marginally statistically significant (OR = 1.68, 95% CI = 1.07–2.63); however, when analyzed by exposure quartile in the analysis that adjusted for other PFAS, none of the individual exposure quartiles (Q4 OR = 2.19, 95% CI = 0.86–5.61) nor the dose response trend (*p* = 0.13) were reported to be statistically significant.
- Winquist et al. (13) is a case–control study conducted in the American Cancer Society’s prospective Cancer Prevention Study II (CPS-II) LifeLink cohort. The authors did not report a statistically significant association between PFOA serum concentrations and kidney cancer in men or women (HR = 1.06, 95% CI = 0.83–1.35) However, when cases were restricted to renal cell carcinoma (RCC), for women only, a statistically significant association was reported (Overall HR = 1.54, 95% CI = 1.05–2.26; Q4 HR = 3.14, 95% CI = 1.04–9.54). Nevertheless, when the authors utilized a statistical model with collapsed categories of some control variables, the association between RCC and PFOA among women was attenuated (i.e., was no longer statistically significant (*p* = 0.055)), indicating small sample size may be impacting the results (as seen in wide confidence intervals for the individual exposure quartile results). This study also did not statistically control for exposure to other PFAS compounds.
- Rhee et al. (14), the same investigators from Shearer et al. (12), conducted a follow-up analysis in a “larger and more racially and ethnically diverse population” to try and replicate their original findings. The authors conducted a nested case–control study in the Multiethnic Cohort Study (MEC), which controlled for multiple appropriate confounders. The authors did not observe any statistically significant associations between PFOA pre-diagnostic serum levels and RCC in their overall analysis (Continuous OR = 0.89, 95% CI = 0.67–1.18; or Q4 OR = 1.04, 95% CI = 0.60–1.81), or in any of the stratified subgroup analyses (by sex, by race, by age, by location, etc.). This finding conflicts with some of their previous results reported in Shearer et al. (12).

In addition, there have been studies that look at impacts from PFAS as a group (rather than specifically to PFOA). For example, Li et al. (17), reported no association between PFAS generally (via drinking water, which included PFOA) and risk of kidney cancer; however, results based on residence alone must be interpreted carefully.

The pooled case–control study, Steenland et al. (15), which combined data from Barry et al. (9) and Shearer et al. (12), reported a range of conflicting results depending on how the authors analyzed the data. The study results ranged from PFOA exposure decreasing risk (i.e., risk of kidney cancer was lower with higher levels of PFOA exposure), to no association (i.e., result was not statistically significant), to some results demonstrating an increase in risk (i.e., increase in PFOA exposure could result in an increase in kidney cancer). This study converted their preferred regression’s first slope to an OR of 2.02 (1.45–2.80), though the second slope in this analysis was not significant and would not have a significant OR. This study did not statistically control for exposure to other PFAS compounds.

The meta-analysis, Seyyedsalehi and Boffetta (16), combined results from studies from 2021 and earlier (7–12). The authors reported a PFOA-specific meta-RR of 1.23 (95% CI: 0.99–1.51). While this risk estimate is suggestive of an association, it does not reach statistical significance and any finding of association may be explained by chance. The authors also note that bias and confounding could not be ruled out in their meta-analysis, which they note precludes the use of their meta-RR in a causation analysis. The lack of a statistically significant finding by Seyyedsalehi and Boffetta (16) demonstrates that the majority of the science considered by the 2012 C8 Panel [plus Shearer et al. (12)] does not support a statistically significant association between PFOA exposure and kidney cancer.

**Figure 1:**
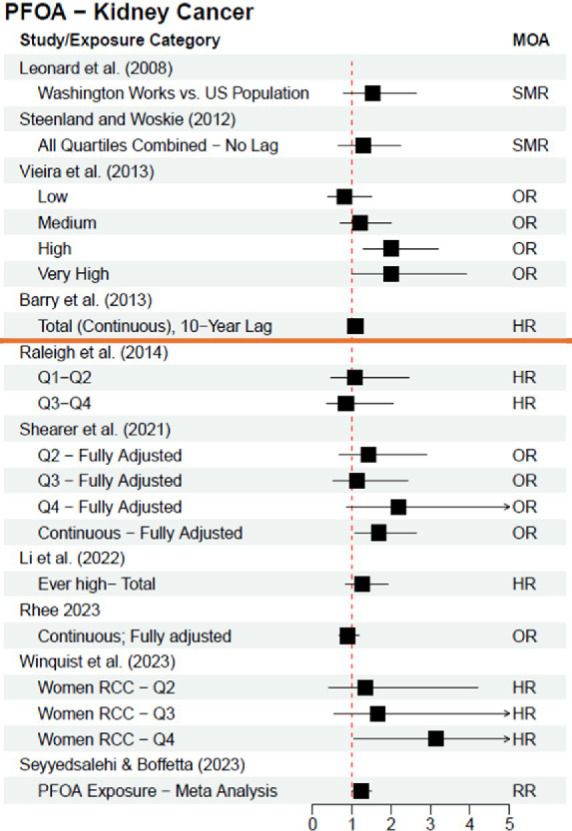
Epidemiological studies investigating the association between exposure to PFOA and risk of kidney cancer; studies above orange line were included in the C8 Panel’s “probable link” report (2012). Leonard et al. (7) results are adjusted from a base 100 SMR. MOA = Measure of Association.

Table 1 outlines the kidney cancer and PFOA studies included in this review, and Figure 1 visualizes the results of the 2012 C8 Panel studies (above the orange line) and the subsequently published studies (below the orange line)—both timeframes clearly demonstrate a lack of consensus and reproducibility across studies; these results do not support a clear causal relationship between PFOA exposure and risk of kidney cancer.

**Table 1:** Descriptions of epidemiological studies included in review of potential associations between PFOA exposure and kidney cancer.

Study	Study design	Study setting	Sample size	Results
Leonard et al. (7)	Cohort	West Virginia, 1948–2002	12 cases	Elevated kidney cancer mortality that was not statistically significantly associated with PFOA exposure.
Steenland and Woskie (8)	Cohort	West Virginia, 1952–2008	11 cases	Increased kidney cancer mortality in highest PFOA exposure quartile, no association observed for cohort overall.
Barry et al. (9)	Cohort	Mid-Ohio Valley, 1952–2011	105 cases	No kidney cancer association observed for cohort overall, highest quartile of community group showed increased risk in unlagged analysis only.
Vieira et al. (10)	Case–control	Ohio and West Virginia, 1996–2005	94 cases	“High” exposure quartile was significantly associated with kidney cancer, “very high” quartile was borderline significant. One out of six municipalities significantly associated.
Raleigh et al. (11)	Cohort	Minnesota, 1960–2008	6 cases	No kidney cancer association observed for cohort overall or in high exposure group.
Shearer et al. (12)	Case–control	10 U.S. cities, 1993–2001	324 cases	Significant association observed in overall cohort with a non-significant p-trend value. In fully adjusted model, no quartiles of PFOA exposure were significantly associated with kidney cancer.
Steenland et al. (15)	Pooled case–control	See contributing studies	835 cases	Multiple regression results with some significantly positive, not significant, and significantly negative results.
Winquist et al. (13)	Case-cohort	USA, 1982–2017	156 cases, 107 cases	No kidney cancer association in overall cohort, a statistically significant association was reported for RCC among women only.
Rhee et al. (14)	Case–control	Hawaii and California, 1993–2018	428 cases	No statistically significant association with PFOA exposure in overall cohort or in stratified analysis.
Seyyedsalehi and Boffetta (16)	Meta-analysis	See contributing papers	–	Suggestive association that is not statistically significant.

The results of studies investigating PFOA exposure and kidney cancer are inconclusive and equivocal (see Figure 1), which highlights the intricacies of study design essential for high quality PFAS epidemiological studies. Differing statistical approaches within studies have resulted in different conclusions. The results may also vary depending on how a study classifies PFOA exposure, incorporates control for confounders, and utilizes different statistical methods. For example:

- Exposure classification: Utilizing pre-diagnostic serum samples versus job exposure matrices or residential address for exposure proxy [e.g., Rhee et al. (14) vs. Vieira et al. (10) and Barry et al. (9)]
- Confounder Control: Controlling for appropriate confounders versus not controlling for a full suite of confounders [e.g., Rhee et al. (14) vs. Barry et al. (9), and Shearer et al. (12) vs. Vieira et al. (10)]
- Statistical Methods: Categorizing exposure as binary or in quantiles based on distribution vs. analyzing as continuous exposure [e.g., Shearer et al. (12) quartile analysis vs. continuous analysis] and stratifying data by sex, race and other population characteristics [e.g., Shearer et al. (12) vs. Rhee et al. (14)].

Earlier studies may not provide analysis or adjustments that by today’s standards are the hallmarks of a “strong” study design. Importantly, no study design is flawless, and studies with limitations (though less useful in assessing causation) provide valuable information regarding the consequence and magnitude of not making proper adjustments.

### Thyroid disease

In 2012, the C8 Science Panel determined that the available epidemiologic data presented sufficient evidence to conclude that there is a “probable link” between PFOA exposure and thyroid disease “despite inconsistencies in the evidence.” (36) “Thyroid disease” is more complicated to evaluate than other outcomes such as kidney cancer because there are various biomarkers of impaired or disrupted thyroid function. There are studies that investigate the potential impact of exposure to PFAS (and to PFOA specifically) on thyroid hormone levels (e.g., triiodothyronine, T3; thyroxine, T4) and thyroid-stimulating hormone (TSH), and also on diagnosed specific thyroid diseases (e.g., hypothyroidism or hyperthyroidism). Many factors can influence an individual’s thyroid hormone levels including autoimmune diseases, genetic factors, thyroid surgery, radiation therapy, different medicines, thyroiditis, pregnancy, iodine intake levels, and smoking, among others (18, 38, 29), which can make it difficult to investigate potential other causal associations. The C8 Science Panel considered 10 epidemiological studies prior to releasing their report (three of which were conducted by the C8 Science Panel), which utilized different analytical approaches and investigated different subsets (i.e., stratifications) of community and occupational populations. The C8 Science Panel noted, “While each finding in isolation was not compelling, plausibly a result [sic] of chance or other errors, the presence of some independent pieces of evidence indicative of an association was not easily dismissed, despite a lack of coherence among them” (36). For sake of brevity, these studies are summarized in Table 2.

**Table 2:** Descriptions of epidemiological studies included in review of potential associations between PFOA exposure and thyroid disease.

Study	Study design	Study setting	Sample size	Results
Melzer et al. (29)	Cross-sectional	USA, 1999–2006	292 cases	Females and thyroid disease: Statistically Significant association with PFOA.
Chan et al. (30)	Case–control	Edmonton, Canada, 2005–2006	11 cases	Pregnant women with hypothyroxinemia: No association with PFOA.
Olsen et al. (31)	Cross-sectional	Cottage Grove, MN, 1993 and 1995	191 exposed	Occupational exposure: No association between TSH and PFOA.
Olsen et al. (32)	Longitudinal and cross-sectional	Belgium and Alabama, 1995–2000	347 exposed	Occupational exposure: PFOA positively associated with T3 increases (within clinical reference range); negative association with FT4 in male workers (within clinical reference range); no association with other hormones.
Bloom et al. (33)	Cross-sectional	New York, 1995–1997	31 exposed	Community of anglers: No association between PFOA and TSH or FT4.
Emmett et al. (34)	Cross-sectional	Ohio and West Virginia, Unspecified time period	371 exposed	Exposed residents: No association between PFOA and TSH levels.
Knox et al. (35)	Cross-sectional	Ohio and West Virginia, 1961–2006	52,296 exposed	Association between TT4 and PFOA (particularly among women), no association with TSH.
Science Panel Study #1	Survival analysis and prospective	Ohio and West Virginia, 2008–2011	3,633 cases	Functional thyroid disease: Mixed results depending on strata.
Science Panel Study #2	Cross-sectional and longitudinal	Ohio and West Virginia, 2005–2006	50,680 exposed	No relationship between PFOA and FT4, TPO, or Tg; no changes in TT4, TSH, or FTI.
Science Panel Study #3	Cohort and cross-sectional	Ohio and West Virginia, 2005–2006	10,725 exposed	Childhood thyroid disease and function: Positively associated with PFOA serum levels in children. No association found between hormones and PFOA levels.

Since the release of the C8 Science Panel report in 2012, there have been multiple cross-sectional community studies investigating potential associations between thyroid diseases and changes in thyroid hormones following exposure to PFOA [e.g., (19–24)]. While some of the results of the studies are conflicting, the vast majority demonstrate no association between PFOA exposure and changes in thyroid hormones or with thyroid disease. However, due to the study design, cross-sectional studies are inherently weaker regarding the evaluation of causation due to the chance of reverse causation (i.e., temporality cannot be determined).

**Figure 2:**
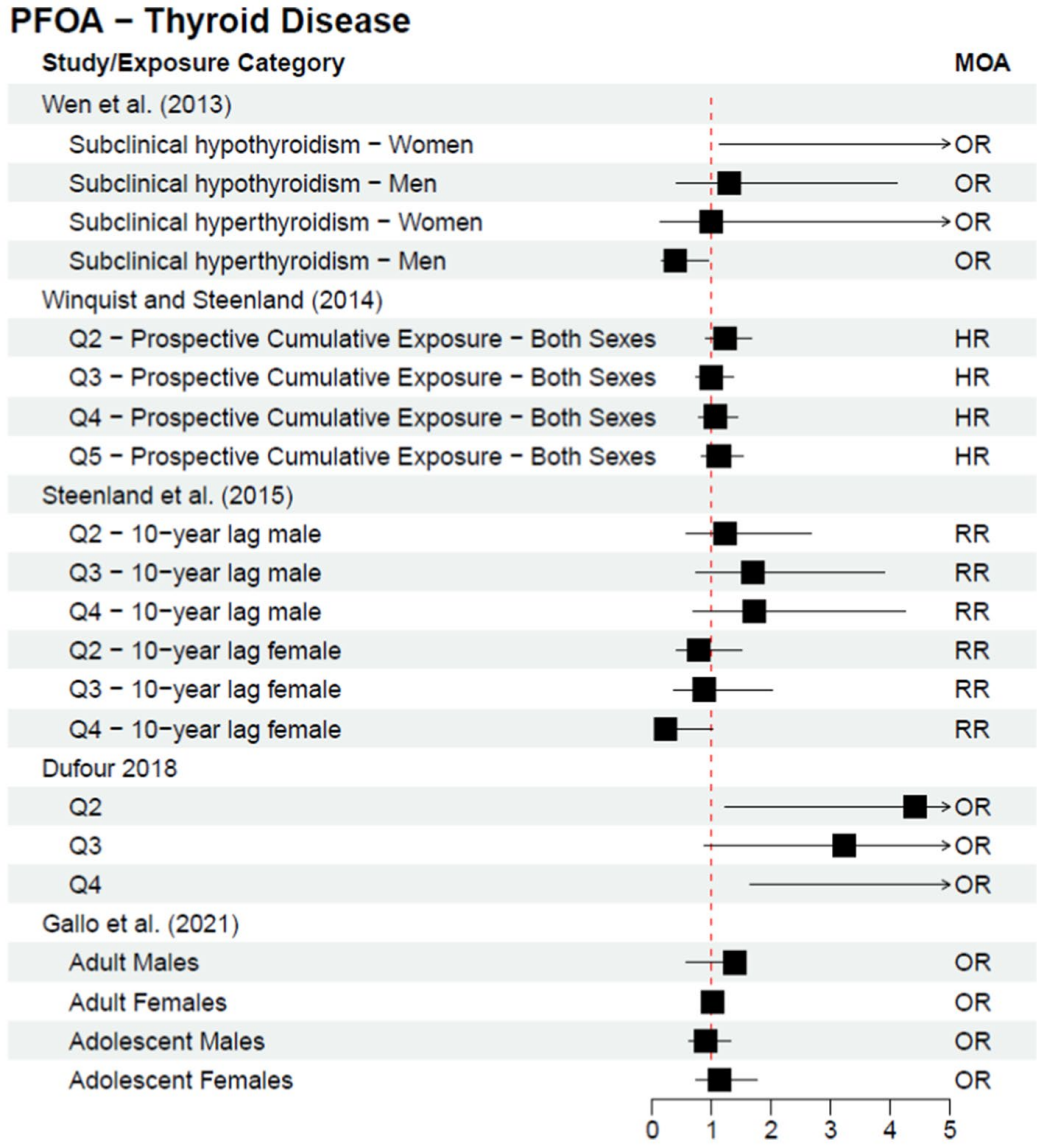
Epidemiological studies published after the C8 Science Panel’s “probable link” report (2012) investigating the association between exposure to PFOA and risk of thyroid disease, reported as risk effect estimates.

**Figure 3:**
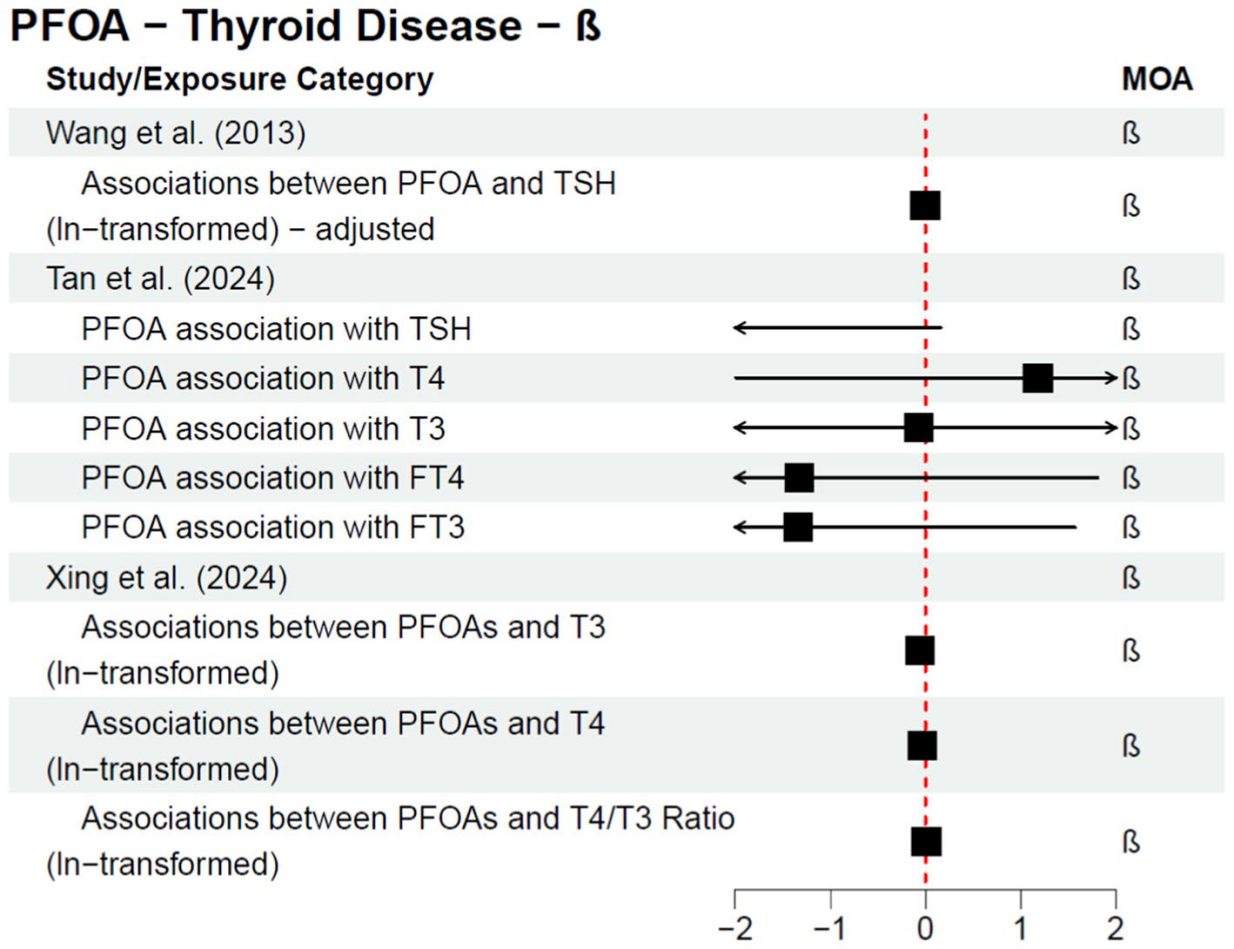
Epidemiological studies published after the C8 Science Panel’s “probable link” report (2012) investigating the association between exposure to PFOA and risk of thyroid disease and thyroid hormone changes, reported as regression coefficients.

Cohort and case–control studies are more useful study designs to inform a potential causal relationship; within these studies, exposure is known to occur before disease diagnosis. One follow-up cohort study, Steenland et al. (25), was published after the C8 Science Panel’s report in 2012 as a continuation of their analysis. The authors stratified analyses by sex and investigated both a 10-year lag and no lag for thyroid disease—all statistical analyses resulted in no statistically significant association for any of the exposure quartiles and/or dose response trend tests. The results from the studies discussed above (a subset of the literature published since the 2012 C8 Science Panel) are included in Figure 2 or Figure 3, depending on the type of measure of association reported within the study; Figure 2 provides risk estimates such as odds ratios, hazard ratios, and risk ratios, with a null value of 1, while Figure 3 provides results reported as beta coefficients from regression models, with a null value of 0. Both figures visually demonstrate that key epidemiological literature published since the 2012 C8 Science Panel report a lack of an association between PFOA exposure and thyroid disease.

Additionally, in 2020, former C8 Science Panel members and collaborators published a review, Steenland et al. (26), commenting on the state of PFOA literature regarding thyroid disorders, cancer, and other health outcomes previously investigated. The authors conclude that, “since the original Science Panel findings, our view is that the evidence of an association of PFOA and thyroid disease has gotten weaker” and that “while a number of studies have suggested associations between thyroid hormones and PFOA in cross-sectional analyses, in our view there is little consistency across studies so evidence for a causal impact on thyroid hormones remains weak.”

More recent epidemiological literature has clarified the conflicting results discussed in the C8 Science Panel’s 2012 report to indicate a lack of an association between PFOA exposure and risk of thyroid disease. As such, it is essential to look at literature beyond the C8 Science Panel, as the results of the C8 Science Panel’s analysis are not aligned with the totality of the body of literature available in 2025. In addition, more recent studies have identified specific subpopulations for future research (e.g., pregnant women) and the importance of investigating individual PFAS rather than the class of compounds together.

## Discussion

Overall, the science around PFAS is rapidly expanding—with the C8 Science Panel’s findings in 2012 acting as a catalyst for further scientific inquiry. Over a decade later, despite prior suggestions of a probable link between PFOA exposure and kidney cancer or thyroid disease, recent epidemiological investigations support that there is no clear causal relationship between PFOA exposure and either health outcome. Specifically:

1. The C8 Science Panel’s “probable link” determination is different from a scientific determination of general causation.
2. At the time of the 2012 C8 Panel, there were only a handful of epidemiological studies that provided the basis for their preliminary determination of a “probable link” focusing on the Mid-Ohio Valley population from the class action settlement. These studies at the time did not support general causation.
3. Additional epidemiological studies conducted since the 2012 C8 Panel reinforce the lack of a causal relationship between exposure to PFOA and kidney cancer or thyroid disease based on the lack of reproducible findings and temporality in well-conducted epidemiological studies.
4. These more recent epidemiological studies reflect more reliable scientific investigations where improvements in exposure classification, confounding control, and statistical methods were incorporated. Therefore, the more recent epidemiological findings provide added robust support for no causal relationship between PFOA exposure and kidney cancer or thyroid disease.

In the past several decades, the scientific understanding of PFAS has advanced in a variety of subject areas (chemistry, mechanistic toxicology, fate and transport, epidemiological research, etc.). Regarding epidemiological studies, this evolution has demonstrated the importance of specificity (regarding both PFAS exposure and disease classification), the need for proper control of confounders, and appropriate statistical stratifications when investigating potential relationships between PFOA (and PFAS) and different health outcomes. Overall, the highest quality epidemiological investigations have demonstrated the importance of incorporating and/or considering the following:

- In terms of exposure,
- It is necessary to analyze for and consider additional PFAS compounds beyond PFOA due to differences in toxicity and toxicokinetics; and
- Exposure classification must continue to be refined (e.g., move beyond utilizing residence as a proxy for exposure), incorporating pre-diagnostic serum sampling with serum half-lives, in addition to retrospective modeling of PFAS exposure via different pathways (e.g., occupational and community).
- In terms of statistical analysis,
- It is critical to control for an appropriate suite of confounders and other chemicals potentially correlated with PFAS;
- When sample size allows, researchers should include stratifications by sex, race, age, and pregnancy status; and
- Disease outcome stratification should be included, such as restricting to renal cell carcinoma rather than all kidney cancer diagnoses or restricting to hypothyroidism rather than all thyroid diseases.

In general, when considering epidemiological evidence, each study will have strengths and weaknesses, which is why consistency and reproducibility are so important when evaluating potential causal relationships. Traditional causation approaches, such as weight of evidence approaches, should incorporate and consider all epidemiological studies, evaluating a study’s quality and weighting stronger, more robust studies over flawed studies. In particular, more weight should be given to studies that demonstrate temporality and thus can demonstrate causation (e.g., prospective cohort studies), as compared to those that can only inform on potential association (e.g., cross-sectional studies). Ultimately, as demonstrated above, the entire body of literature needs to be included in this process to reflect the evolution of the scientific process. The C8 Science Panel’s 2012 “probable link” report was conducted under the context of litigation, specific to the class members and exposure to high levels of PFOA; due to the methodology used, the “probable link” conclusions were distinct from general causation assessments.

Regulatory agencies have also recently performed evaluations of epidemiological evidence. Although the recent IARC evaluation (27) classified PFOA as a Group 1 carcinogen, the Working Group concluded that evidence in humans for the association between PFOA and renal cell carcinoma was “limited.” Similarly, the USEPA (28) classified PFOA as “Likely to Be Carcinogenic to Humans” but noted there is “not [a] definitively causal association between human exposure to PFOA and cancer outcomes.” These regulatory evaluations are performed for purposes and in contexts different than general causation assessments and weigh mechanistic and animal data differently depending on the agency. Therefore, the purpose, protocols, limitations, and context of regulatory determinations need to be understood—especially when trying to assess potential for actual disease manifestation in exposed individuals.

In conclusion, this evaluation distinguishes between legal rulings and settlements and scientific causation, dispelling the notion that legal rulings equate to “settled science.” The C8 Science Panel was established as part of a settlement and its determinations, per the terms of the settlement, were intended to help resolve what claims in that litigation would be compensated and which ones would not. The C8 Science Panel’s “probable link” determinations are often confused with a scientific determination of general causation. Despite the “probable link” determinations, the available epidemiological studies at the time did not support general causation for kidney cancer or thyroid disease. While this review focused on human epidemiology studies, it is important to be mindful of the existence of related *in vitro* and animal bioassays; a full review for establishing causation would also take full account of the available mechanistic and animal model evidence. As well, research into PFAS health effects is a highly active area of study, so the emergence of new epidemiologic evidence may change the weight of evidence view of the science. The evolution of PFOA epidemiological literature since the 2012 C8 Science Panel demonstrates how science is dynamic—despite prior suggestions of a “probable link” between exposure PFOA and kidney cancer or thyroid disease, recent epidemiological investigations support that there is no clear causal relationship between PFOA exposure and either health outcome.

## Footnotes

^1^Aqueous Film-Forming Foams (AFFF) Products Liability Litigation, MDL No. 2873, U.S. District Court for the District of South Carolina.
